# Fasciocutaneous anterolateral thigh flaps for complex abdominal wall reconstruction after resection of enterocutaneous fistulas and the role of indocyanine green angiography: a pilot study

**DOI:** 10.1007/s10029-020-02167-w

**Published:** 2020-03-26

**Authors:** T. Pruimboom, I. B. M. Ploegmakers, E. Bijkerk, S. O. Breukink, R. R. W. J. van der Hulst, S. S. Qiu

**Affiliations:** 1 Department of Plastic and Reconstructive Surgery, Maastricht University Medical Center, P. Debyelaan 25, 6229 HX Maastricht, The Netherlands; 2 Department of Surgery, Maastricht University Medical Center, P. Debyelaan 25, 6229HX Maastricht, The Netherlands

**Keywords:** Enterocutaneous fistula, Abdominal wall reconstruction, ALT, ICG

## Abstract

**Purpose:**

No previous study reported the use of a fasciocutaneous anterolateral thigh (ALT) flap combined with a biological mesh for abdominal wall reconstruction (AWR) after enterocutaneous fistula (ECF) in a single-staged procedure and the use of Indocyanine Green Angiography (ICGA) intraoperatively. The purpose of this study was to determine the feasibility and safety of this procedure and to examine the added value of ICGA in minimizing postoperative complications.

**Methods:**

A single-institution review of a prospectively maintained database was conducted at Maastricht University Medical Center. To evaluate the feasibility and safety of this procedure, early (≤ 30 days) and late (> 30 days) postoperative complications were assessed. ECF recurrence was considered the primary outcome. To examine the added value of ICGA, complications in the ICGA group and the non-ICGA group were compared descriptively.

**Results:**

Ten consecutive patients, with a mean age of 66.7 years, underwent a single-staged AWR with fasciocutaneous ALT flaps. Mean follow-up was 17.4 months (4.3–28.2). Two early ECF recurrences were observed. Both restored without the need for reoperation. A lower rate of early complications was observed in the ICGA group compared to the non-ICGA group.

**Conclusion:**

The combination of a biological mesh and fasciocutaneous ALT flap is feasible and safe in AWR after ECF repair in a single-staged approach, with an acceptable complication rate in a cohort of complex patients operated in a dedicated center. ECF closure was achieved in all patients. ICGA seems to be of great added value in minimizing postoperative complications during AWR.

**Electronic supplementary material:**

The online version of this article (10.1007/s10029-020-02167-w) contains supplementary material, which is available to authorized users.

## Purpose

An enterocutaneous fistula (ECF) is defined as an unnatural communication between the gastrointestinal tract and the skin. ECFs allow intestinal contents to leak and can result in electrolyte abnormalities, fluid imbalances, malnutrition and sepsis [1]. Conservative management alone, including optimal nutritional and metabolic support, wound care and antimicrobial therapy, results in ECF closure of approximately one-third of the cases over a period of three to six months [1, 2]. Lower fistula output volume (low < 200 ml/day, moderate 200–500 ml/day and high > 500 ml/day), is associated with a higher chance of closure [1, 2].

In case of a persistent ECF, surgical intervention is needed. The purpose of surgery is to resect the fistula complex, which includes the bowel segment giving rise to the fistula, re-establishing intestinal continuity and providing definitive abdominal wall reconstruction (AWR). Since ECFs often coexist with major abdominal wall defects [3], creating a new abdominal wall might represent a surgical challenge. Current preferred reconstructive options include component separation and local flaps, combined with or without a biological mesh to create coverage of the abdominal cavity [3, 4]. A biological mesh consists of an extracellular collagen matrix that is gradually replaced with a collagen framework. This allows for cellular regeneration, neovascularization and remodulation of the mesh into a neofascia which should withstand mechanical forces. Evidence suggests that biological meshes are less sustainable and are associated with higher abdominal hernia and bulging rates when compared to synthetic material [4]. However, biological meshes are associated with higher salvage rates in cases of infection [5]. Hernia and fistula recurrence rates reaching up to 41.7% have been reported after component separation techniques in combination with biological mesh [6]. Moreover, component separation may not be sufficient to restore abdominal integrity in large abdominal wall defects. Therefore, autologous tissue flaps are recommended in these cases [4, 7–9].

Anterolateral thigh (ALT) flaps have become increasingly popular in reconstructive surgery since their use was first reported in 1984 [10]. Although the ALT flap is well described and is found to be a feasible autologous tissue flap for reconstruction of various abdominal defects [11–14], there is a paucity in the literature concerning ALT flaps in AWR after ECF resection [3, 7, 15–17]. Since a biological mesh has the advantage of reconstructing abdominal fascia in a contaminated area and an ALT flap has the advantage of restoring a complex and large abdominal wall defect, the combination of both seems feasible in these complex ECF cases. The combination of a musculocutaneous ALT flap and a biological mesh has recently been reported in a single-staged AWR after ECF resection [3]. In our opinion, a fasciocutaneous ALT flap combined with a biological mesh, instead of a musculocutaneous ALT flap seems a more attractive reconstructive option, taking into account the lower probability of donor-site morbidity. To the best of our knowledge, no study reported the use of a fasciocutaneous ALT flap and a biological mesh in single-staged surgery.

To minimize postoperative complications, it is important to assess tissue perfusion intraoperatively as immediate debridement of insufficiently perfused portions of both the ALT flap and abdominal skin edges might reduce postoperative wound complications. Current evaluation of tissue perfusion depends highly on the surgeon’s experience and is found to be an unreliable predictor of perfusion [18]. Therefore, the use of Indocyanine Green Angiography (ICGA) is suggested to improve postoperative outcomes in flap surgery [19]. ICGA uses Indocyanine Green (ICG) as a contrast dye with fluorescent characteristics. After intravenous administration, ICG is bound to plasma protein. When exposed to near-infrared excitation, it reemits fluorescent light, which enables in-depth visualization of blood vessels. This innovative technique allows for real-time imaging of tissue perfusion and aids in performing accurate excision of non-vital tissue intraoperatively [18, 19].

The purpose of the current study was twofold. First, to determine the feasibility and safety of single-stage AWR using a fasciocutaneous ALT flap combined with a biological mesh after ECF resection and second, to examine the added value of ICGA in minimizing postoperative complications.

## Methods

A single-institution review of a prospectively maintained database was conducted on ten consecutive patients who underwent single-staged ECF repair and AWR with a fasciocutaneous ALT flap at Maastricht University Medical Center between July 2017 and September 2018. All procedures performed in this study were in accordance with the ethical standards of the institutional research committee (Medical Ethics Committee in Maastricht: METC 2018-0941) and with the 1964 Helsinki Declaration and its later amendments or comparable ethical standards. All ALT flaps were combined with a non-cross-linked porcine-derived biological mesh (Strattice^™^, LifeCell Corp., Branchburg, NJ, USA).

Preoperative patient characteristics were obtained from the medical records of the included patients and from a prospectively maintained database from the Department of Plastic and Reconstructive Surgery. Variables obtained included age, Body Mass Index (BMI), smoking status, diabetes, previous abdominal surgeries, ECF classification and time from ECF onset to surgery. Surgical characteristics, including free or pedicled ALT flap, operating time, hospital stay, and follow-up were registered. To evaluate feasibility and safety of this procedure, postoperative complications were assessed. Complications were categorized into early (within 30 days following surgery) and late (after 30 days) complications.

ECF recurrence was considered the primary outcome, proven by the clinical presence of fistula fluid, or confirmed by a computed tomography (CT) scan. Secondary outcomes included abdominal hernia and bulging, flap loss, partial flap necrosis, wound dehiscence, mesh exposure, surgical site infection (SSI), seroma and hematoma. Abdominal hernia was defined as dehiscence of the fascial closure, confirmed by a CT scan and abdominal bulging was defined as any asymmetrical abdominal bulge. Flap loss was defined as the removal of the ALT flap and partial flap necrosis was defined as loss of a part of the flap. Wound dehiscence was defined as any wound rupture over the incision line. Mesh exposure was defined as exposure of (part of) the mesh. We defined SSI as documented erythema, abscess, or purulent drainage requiring treatment with antibiotics or surgical intervention, regardless of whether positive cultures were obtained. Seromas were defined as a fluid collection, verified and treated by ultrasound-guided needle puncture. A hematoma was defined as any collection of blood with or without the need for intervention.

Patients were split into two groups: the ICGA and non-ICGA group. In the non-ICGA group, perfusion of both the ALT flap and abdominal skin edges were clinically evaluated by the plastic surgeon, by determining color and temperature of the skin, capillary refill and dermal edge bleeding. In the ICGA group, intraoperative ICGA was performed with Fluobeam^™^ (Fluoptics, Grenoble, France) to evaluate perfusion. In both groups, excision of ALT flap and abdominal skin edges was performed when perfusion seemed insufficient. Rates of postoperative complications in the two groups were described.

Continuous variables were reported as mean with standard deviation and minimum and maximum for normally distributed data and reported as median with minimum and maximum for not normally distributed data. Categorical data were reported as frequencies and proportions. Data were analyzed using IBM SPSS Statistics for Mac, version 25 (IBM Corp., Armonk, NY, USA). We decided not to perform statistical analysis to compare patients and surgical characteristics, and complications between the ICGA and non-ICGA group, due to a lack of statistical power in this small sample size.

### Surgical procedure

The general surgeon, specialized in gastrointestinal surgery, started the procedure. First, extensive adhesiolysis was performed to detect and expose the ECF. The fistula complex, including all bowel segments involved, was resected. Hereafter, intestinal continuity was re-established with a side-to-side stapled anastomosis, and the complete intestine was screened for unnoticed serosal injuries.

**Fig. 1:**
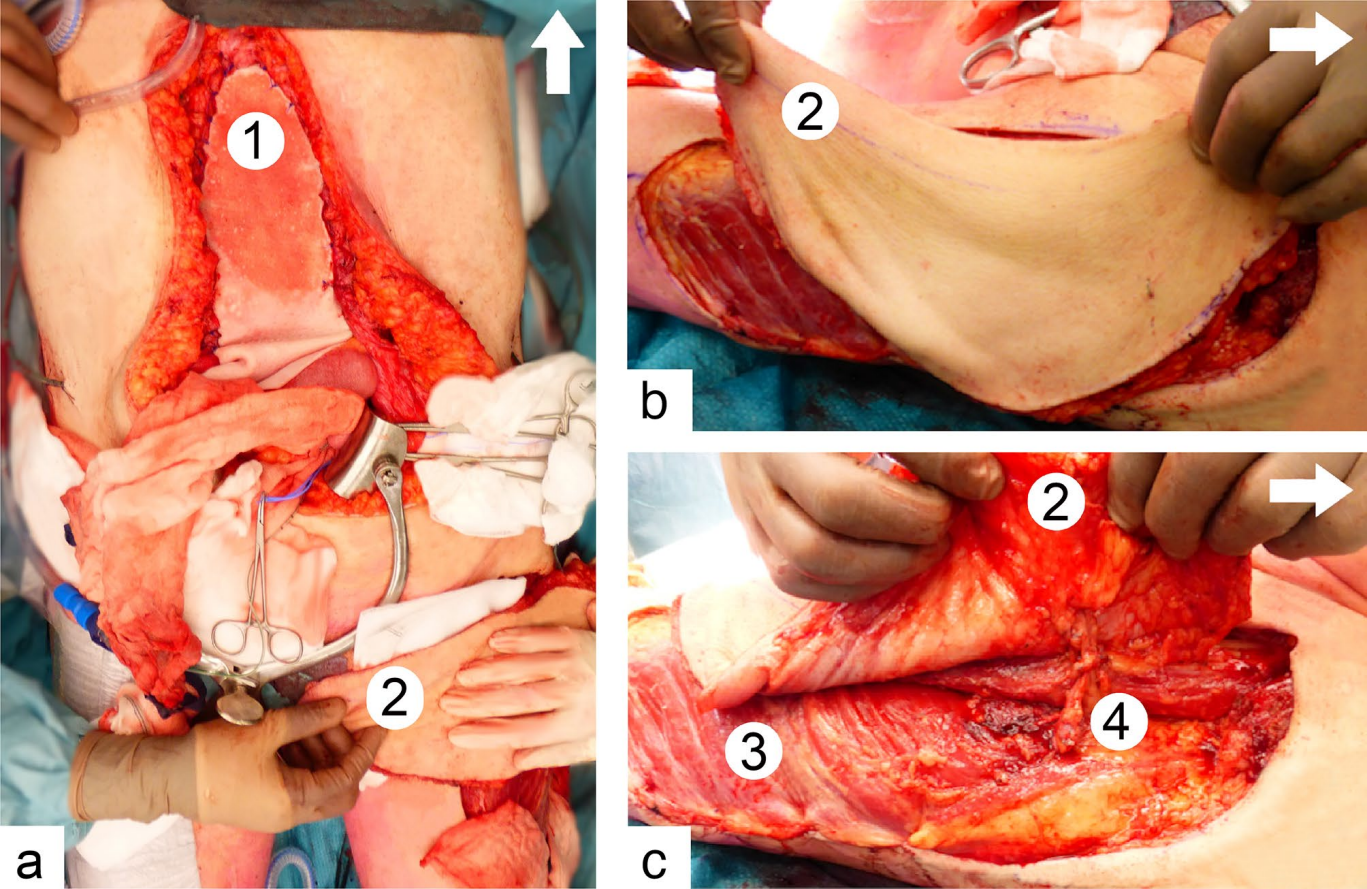
Intraoperative overview of the abdominal wall reconstruction (arrow indicating the cranial side). **a** Intraoperative aspect of the abdominal wall after suturing the biological mesh (1) to reconstruct the fascial defect and the fasciocutaneous ALT flap (2) that was harvested. **b** Intraoperative aspect of the fasciocutaneous ALT flap with intact underlying musculus vastus lateralis (3). **c** ALT flap raised on its vascular pedicle (4)

After resection of the ECF complex, the biological mesh 20 × 30 cm in size was cut to the defect size, positioned underlay with an overlap of at least 4 cm and fixed to the remaining the abdominal musculofascial layer with interrupted Prolene^®^ Polypropylene sutures (Ethicon Inc. Somerville, NJ, USA) size 2-0 in an outer and inner ring to reconstruct the fascia of the rectus abdominis muscles in all patients. See Fig. 1a.

Following fascial closure, a free or pedicled ALT flap was harvested as a fasciocutaneous flap and raised on the descending branch of the lateral femoral circumflex artery. See Fig. 1b, c. In case of a pedicled ALT flap, the flap was tunneled under the rectus femoris muscle to reach the defect in the abdomen.

**Fig. 2:**
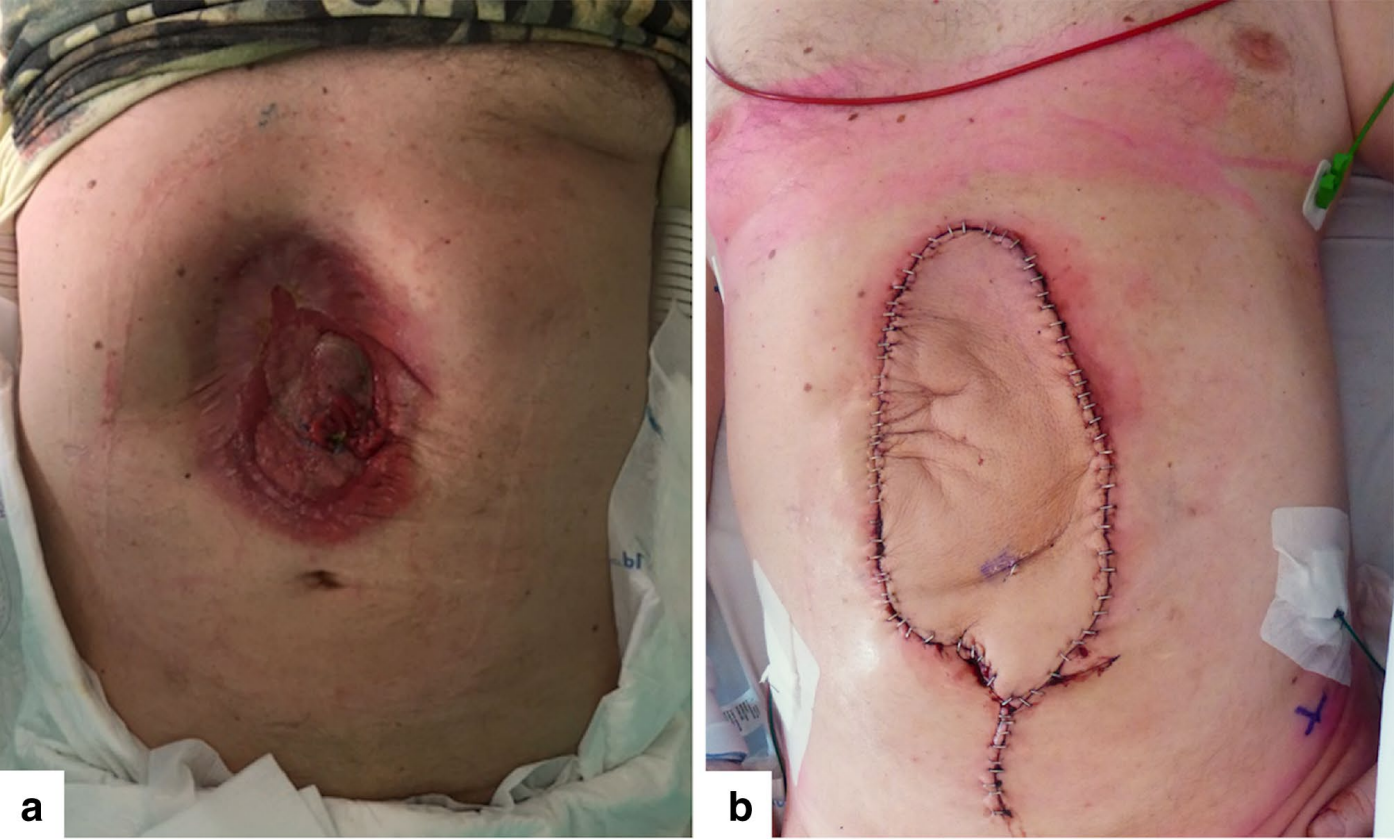
**a** Preoperative aspect of the abdominal wall. **b** Postoperative result of the abdominal wall reconstruction

In the case of a free ALT flap, a pedicle of approximately 10–12 cm was harvested and the anastomosis was performed underneath the rectus abdominis muscle to the inferior epigastric vessels [20]. The size of the flap was determined according to the defect of the abdominal wall after suturing the biological mesh. For the defects at the level of the  umbilicus or lower, a pedicled flap was used whereas for defects located more cranially, a free ALT flap was preferred. The fascia and subcutaneous tissue of the ALT flap were sutured to the remaining muscular fascia of the abdominal wall. The skin island of the ALT flap was placed above the biological mesh, covering it in its totality. The abdominal skin was sutured to the skin island of the ALT flap, after de-epithelization of the edges of the flap. See Fig. 2a, b for the preoperative and postoperative aspect of the abdomen, respectively. The donor-site wound on the anterolateral thigh was approximated on both the cranial and caudal side without undue tension, and the remaining defect was covered with a split skin graft.

### ICGA protocol

Vascularization of the ALT flap and abdominal skin edges were checked after flaps harvesting. The handheld imaging-head was manually positioned approximately 20–25 cm above the skin. Real-time grayscale video-angiography was obtained for 120 s, starting 10 s after intravenous injection of 0.1 mg/kg of ICG (Diagnostic Green, Aschheim-Dornach, Germany).

Fluorescent regions (i.e., white area) indicated areas of well perfused, whereas no fluorescence (i.e., black area) and less fluorescence (i.e., gray area) indicated areas of insufficiently perfused tissue and these were removed during surgery. No additional relative perfusion assessment or absolute flow value assessment using software were used. See Online Resource 1 for an intraoperative ICG video angiography with a description of the images over time.

## Results

### Patient characteristics

A total of ten consecutive patients underwent single-staged AWR with a fasciocutaneous ALT flap combined with biological mesh after ECF resection. Table 1 summarizes the main characteristics of the sample size. Median follow-up was 17.4 months (4.3–28.2). One patient died by suicide 4.3 months after surgery. This patient was known with psychiatric complaints that were not related to the abdominal wall reconstruction. Follow-up measurements of this patient were excluded from the ‘late complication’ analysis. Two patients were operated a second time within the first 30 days after initial surgery to revise the arterial anastomosis (*n* = 1) and to explore a suspected bowel leakage (*n* = 1).

**Table 1:** Patient and surgical characteristics

Variable	Statistics
Total patients	10
Age (years)	
Mean (SD)	66.7 (9.6)
Min–max	53–78
Body Mass Index (kg/m^2^)	
Mean (SD)	26.4 (4.7)
Min–max	19.3–35.3
Diabetes, *n* (%)	4 (40)
Smoking, *n* (%)	0 (0)
Previous surgeries	
Median^a^	4
Min–max	2–15
ECF classification, ^b^ *n* (%)	
Low	5 (50)
Moderate	2 (20)
High	3 (30)
Fistula onset to surgery (days)	
Mean (SD)	185 (83)
Min–max	56–313
ALT, *n* (%)	
Free	7 (70)
Pedicled	3 (30)
Operating time (min)	
Mean (SD)	634 (89)
Min–max	434–780
Hospital stay (days)	
Median^b^	28
Min–max	14–105
Follow-up (months)	
Mean (SD)	17.4 (7.3)
Min–max	4.3–28.2

^a^The data from previous surgeries and hospital days were not normally distributed

^b^Low output < 200 ml/day, moderate output 200–500 ml/day, high output > 500 ml/day [1]

### Primary outcome

ECF recurrence was observed in two patients at day 14 and day 17 after surgery, respectively. Both were proven by the clinical presence of fistula fluid and classified as low output fistulas. ECF closure was achieved with conservative treatment according to the SOWATS guidelines [21], after 4 days and 8 weeks, respectively. Consequently, ECF closure rate was 100% without the need for reoperation during follow-up.

### Secondary outcomes

One patient was diagnosed with an abdominal hernia confirmed by a CT scan, 3 months after surgery. Abdominal bulging was observed in three patients. None of these cases required reoperation as symptoms of discomfort and abdominal pain dissolved by wearing abdominal binders.

No ALT flaps were lost. Early partial ALT flap necrosis occurred in two patients. In one patient, necrosis was first observed 2 days after surgery. The necrotic part was debrided in the patient ward, followed by vacuum-assisted wound therapy. In the other patient, necrosis was observed 18 days after surgery. After debridement and vacuum-assisted wound therapy, the remaining defect was closed with a rhomboid flap 66 days after surgery.

Wound dehiscence was observed in five patients (four early and one late cases). Two out of five patients experienced wound dehiscence following ALT flap necrosis as described above. The remaining three patients were treated with conservative wound therapy with (*n* = 2) or without (*n* = 1) vacuum-assisted therapy. Mesh exposure as a result of wound dehiscence was observed in two out of six of the aforementioned patients. The biological mesh was covered with granulation tissue in both patients in a few days and no patient required explantation of the biological mesh.

Six patients experienced an early SSI. One patient was treated with antibiotics only for a superficial SSI and five patients were treated with a combination of ultrasound-guided aspiration and antibiotics for deep SSIs. Three patients experienced early seroma and were treated with ultrasound-guided aspiration, seven and fifteen days after surgery, respectively. No hematomas were observed during this study.

### Indocyanine green angiography

Intraoperative perfusion assessment with ICGA was performed in five patients. ICGA was performed by the plastic surgeon when the ICGA device was available. Table 2 summarizes the main characteristics of patients in the ICGA group and patients in the non-ICGA group. To examine the added value of ICGA, postoperative complications in the ICGA group versus the non-ICGA group were compared. See Table 3.

**Table 2:** Patient and surgical characteristics ICGA versus non-ICGA

Variable/statistics	ICGA	Non-ICGA
Total patients	5	5
Age (years)		
Mean (SD)	66.2 (9.5)	67.2 (10.8)
Min–max	54–77	53–78
Body mass index (kg/m^2^)		
Mean (SD)	26.6 (5.8)	26.1 (3.9)
Min–max	19.3–35.3	21.9–32.1
Diabetes, *n* (%)	1 (20)	3 (60)
Smoking, *n* (%)	0 (0)	0 (0)
Previous surgeries		
Median^a^	4	4
Min–max	2–7	3–15
ECF classification, *n* (%)		
Low	3 (60)	2 (40)
Moderate	1 (20)	1 (20)
High	1 (20)	2 (40)
Fistula onset to surgery (days)		
Mean (SD)	227 (69)	142 (78)
Min–max	146–313	56–221
ALT, *n* (%)		
Free	3 (60)	4 (80)
Pedicled	2 (40)	1 (20)
Operating time (min)^b^		
Mean (SD)	638 (130)	630 (32)
Min–max	434–780	577–658
Hospital stay (days)		
Median^a^	22	32
Min–max	14–105	23–98
Follow-up (months)		
Mean (SD)	14.4 (2.5)	20.3 (9.6)
Min–max	12.6–18.6	4.3–28.2

^a^The data from previous surgeries and hospital days are not normally distributed

^b^Total operation time (i.e., surgical and reconstructive time)

**Table 3:** Early and late postoperative complications in total group, ICGA-group and non-ICGA group

Complication	Total (*n* = 10)	ICGA (*n* = 5)	Non-ICGA (*n* = 5)
Early complications *n* (%)^a^			
ECF recurrence	2 (20)	1 (20)	1 (20)
Partial flap necrosis	2 (20)	0 (0)	2 (40)
Wound dehiscence	4 (40)	1 (20)	3 (60)
Mesh exposure	2 (20)	0 (0)	2 (40)
Infection	6 (60)	4 (80)	2 (40)
Seroma	3 (30)	1 (20)	2 (40)
Any early complication	8 (80)	3 (60)	5 (100)
Total, *n* (% of complications)	19	7 (37)	12 (63)
Late complication, *n* (%)^a^	Total (*n* = 9)^b^	ICGA (*n* = 5)	Non-ICGA (*n* = 4)
Abdominal hernia	1 (11)	0 (0)	1 (25)
Abdominal bulge	3 (33)	2 (40)	1 (25)
Wound dehiscence	1 (11)	1 (20)	0 (0)
Any late complication	5 (56)	3 (60)	2 (50)
Total, *n* (% of complications)	5	3 (60)	2 (40)

^a^Early complication: onset within 30 days following operation. Late complication: onset after 30 days following operation

^b^Follow-up measurements of patient who died by suicide were excluded from the ‘late complication’ analysis

## Discussion

Reconstruction of a large and complex abdominal wall defect after ECF resection is challenging and requires surgical creativity. The combination of an ALT flap and biological mesh is a potential solution to reconstruct large abdominal wall defects with the potential advantage of lowering the chance of ECF recurrence and postoperative abdominal hernia and bulging rates [7, 16]. We reviewed our experience with single-staged AWR after ECF resection, utilizing fasciocutaneous ALT flaps combined with biological mesh, to determine the feasibility and safety in the face of ECF recurrence and other postoperative complications.

Two ECF recurrences have been observed early after the operation, representing a 20% ECF recurrence rate in this study. This is comparable to the reported ECF recurrence rates ranging from 9% to 41.7% in studies concerning ECF repair and AWR with biological mesh reinforcements techniques without the use of ALT flaps [6, 8, 22]. For instance, Connolly et al. reviewed 61 patients who underwent ECF repair and simultaneous AWR with component separation technique. After stratification for closure technique, they found a 41.7% ECF recurrence rate after suture repair, combined with a porcine collagen mesh during a median follow-up time of 29 months [6]. Krpata et al. and Atema et al. reported substantially lower ECF recurrence rates of 10.8% after a mean follow-up of 20 months and 9% after a mean follow-up of 7 months, respectively [8, 22]. However, 25% and 57% of patients who experienced ECF recurrence after ECF repair and AWR in their series, respectively, were in need of reoperation. In the current study, both recurrent ECFs were closed definitively with conservative treatment only. Importantly, no ECF recurrences have been observed during long-term follow-up in any of the included patients.

Likewise, rates of abdominal herniation and bulging ranging from 13% to 50.6% have been reported in previous studies concerning contaminated AWR, with reoperation rates reaching 50% [6, 8, 22–26]. For instance, Kaufmann et al. conducted a multicenter cross-sectional cohort study of 77 patients and reported an abdominal hernia rate of 28.6% and an abdominal bulging rate of 50.6% after a mean follow-up of 22.2 months. Of those patients, 45% of patients underwent reoperation [26]. Although Atema et al. reported a low rate of abdominal hernia of 13%, half of these patients underwent reoperation during follow-up [22]. In our study, lower rates of abdominal hernia and bulging were observed and most importantly, no patient required reoperation. All four patients were treated conservatively by wearing abdominal binders, without experiencing physical complaints.

Single-staged AWR with ECF repair is known to be associated with a high rate of postoperative infections. The SSI rate in our series of complex patients was 60%, which falls within the range of previously described rates following AWR after ECF repair (27.3–65%) [6, 8, 24, 26]. In contrast to the safety of a biological mesh to endure exposure and SSI [8], biological mesh removal rates as high as 6.5% have been reported in recent studies [25, 26]. Although a mesh exposure rate of 20% was observed in the current study, no explantation of a biological mesh was required.

In the current study, we utilized an ALT flap with a biological mesh instead of current preferred reconstructive options (e.g., component separation techniques and local flaps) to create coverage of the abdominal wall. The additive benefit of an ALT flap is that AWR of large defects can be performed without undue tension on the abdominal skin, which would have been inevitable with component separation or local flaps. Another benefit is that the ALT flap is thicker compared to the native abdominal skin and thus provides more strength to the abdominal wall once the biological mesh is remodeled into neofascia over approximately 6 months [27]. The results obtained in this series showed promising (i.e., comparable to better) long-term outcomes in comparison with the existing literature. Consequently, we demonstrated that the combination of a biological mesh and fasciocutaneous ALT flap in AWR in a single-staged procedure is feasible and safe with an acceptable complication rate. During follow-up, no mortality related to the surgical procedure was observed. The majority of complications were considered minor complications since, in most cases, no surgical reintervention was needed after a long follow-up.

The ability to compare our data with other published series concerning ALT flaps in single-staged ECF repair is limited. To the best of our knowledge, this pilot study comprised the first series that aimed to evaluate single-staged AWR using fasciocutaneous ALT flaps combined with a biological mesh after ECF resection. Only few authors have reported their early experience using this procedure [3, 7, 15–17].

Two independent case reports described the use of a pedicled ALT flap to successfully close an abdominal defect after resection of a malignant ECF [16] and to close an abdominal defect with an untraceable ECF [15]. Lambe et al. reported a retrospective review on seven patients who had undergone massive extensive enteroatmospheric fistulation with a pedicled subtotal lateral thigh flap including the vastus lateralis muscle without a biological mesh [17]. Although no ECF recurrences were observed in this study, a higher rate of abdominal herniation of 14% was observed [17]. Walia et al. also reported a retrospective review on seven patients who underwent a pedicled ALT flap for AWR. However, no single-staged ECF repair and AWR were performed since the authors favored a staged approach for ECF patients [7].

More recently, another author reported a series of eighteen patients who had undergone single-staged ECF repair and AWR with a musculocutaneous ALT flap and biological mesh. During a mean follow-up of 24 months, no ECF recurrences were observed and abdominal bulging was observed in only one patient [3]. The lower rate of abdominal hernia and bulging compared to the rates in current study, could be due to the fact that the addition of vastus lateralis included in musculocutaneous flaps might provide extra fascial strength in comparison to a fasciocutaneous ALT flap [3, 7, 17]. On the other hand, musculocutaneous flaps can potentially increase donor-site morbidity [3]. In our experience, there is a low risk of donor-site morbidity after harvesting a fasciocutaneous ALT flap and covering the donor-site defect with a split skin graft. No patient experienced donor-site morbidity in this study. In our opinion, the additive benefit of a biological mesh over fascia or muscle component of an ALT flap is that a mesh is easier to be customized (i.e., cut to size) to cover the size of the defect. Furthermore, a mesh is easier to be fixed in an underlay position in the abdominal defect, before positioning the ALT flap.

ICGA is previously found to be a more reliable predictor of tissue perfusion compared to clinical assessment [28]. In this pilot study, a lower rate of early complications was observed in the ICGA group compared to the non-ICGA group. In particular, rates of perfusion related complications including partial flap necrosis and wound dehiscence were lower in the ICGA group (0–40%, respectively) compared to the non-ICGA group (40–60%, respectively). Resection of insufficiently perfused abdominal skin and portions of the ALT flap in the ICGA group, resulted in a necrosis rate of 0%. This observation is consistent with results reported by Wormer et al. who performed a randomized controlled trial evaluating ICGA in reducing wound complications in complex AWR. Although they found a 6.1% rate of skin necrosis in the non-ICGA group versus 2.2% in the ICGA group, this difference was not statistically significant [29]. In addition to the assessment of abdominal skin and ALT flap perfusion, ICGA serves as an effective tool to assess anastomotic bowel perfusion [30]. Moreover, it could have a role in reducing anastomotic leakage rates following ECF resection, which reflects the potential for implementation of ICGA in reconstructive abdominal wall surgery.

Several limitations of the present study need to be addressed. Due to the limited number of patients included in the current study, no statistical analyses were performed. Furthermore, the retrospective design may have resulted in missing data and selection bias. However, accurate outcome data were entered into a prospectively maintained database so this risk should be minimized. The results of this study cannot be generalized to every hospital, since all surgical procedures are performed in a dedicated center, experienced in abdominal wall surgery and reconstructive surgery. Conversely, this study is strengthened by the fact that that all AWRs are performed by the same team and using the same surgical strategy.

As the number of operated patients increases, more data will become available. In the future we will collect more data on this specific cohort of complex ECF patients to gain more expertise and improve the proposed surgical procedure.

## Conclusion

The combination of a biological mesh and fasciocutaneous ALT flap is feasible and safe in AWR after ECF repair in a single-staged approach, with an acceptable complication rate in a cohort of complex patients operated in a dedicated center. ECF closure was achieved in all patients. Since a lower number of early complications were observed in the ICGA group, ICGA seems to be of great added value. Further research using the same procedure in the context of prospective clinical trials is needed to confirm the role of ICGA in AWR.

## Electronic supplementary material

Below is the link to the electronic supplementary material.

Online resource 1. Intra-operative ICG video angiography during abdominal wall reconstruction. 0:00 to 0:08: start of arterial inflow of fluorescent ICG bound to albumin. 0:09 to 0:19 (cranial up, caudal down): slowly increasing fluorescence intensity during assessment of the abdominal wall). 0:20 to 0:57 (proximal right, distal left): assessment and marking of the ALT flap with a clear difference in fluorescence intensity between the distal and proximal portion of the ALT flap. The region of no fluorescence (i.e., black area) is marked and removed in this patient. 0:58 to 1:42 (cranial up, caudal down): during assessment of the abdominal skin the region of less fluorescence (i.e., gray area) is marked and removed. The remaining abdominal skin is sufficiently perfused, indicated by bright fluorescence regions (MP4 81917 kb)

## References

[1] Whelan JF, Ivatury RR (2011). Enterocutaneous fistulas: an overview. Eur J Trauma Emerg Surg.

[2] de Vries FEE, Atema JJ, van Ruler O, Vaizey CJ, Serlie MJ, Boermeester MA (2018). A systematic review and meta-analysis of timing and outcome of intestinal failure surgery in patients with enteric fistula. World J Surg.

[3] Adabi K, Manrique OJ, Vijayasekaran A, Moran SL, Ciudad P, Huang TCT, Nicoli F, Bishop S, Chen HC (2018). Combined single-stage enterolysis with pedicle seromuscular bowel flaps, myocutaneous and fasciocutaneous flaps to repair recurrent enterocutaneous fistulas in complex abdominal wall defects. Microsurgery.

[4] Latifi R (2016). Practical approaches to definitive reconstruction of complex abdominal wall defects. World J Surg.

[5] Slater NJ, van der Kolk M, Hendriks T, van Goor H, Bleichrodt RP (2013). Biologic grafts for ventral hernia repair: a systematic review. Am J Surg.

[6] Connolly PT, Teubner A, Lees NP, Anderson ID, Scott NA, Carlson GL (2008). Outcome of reconstructive surgery for intestinal fistula in the open abdomen. Ann Surg.

[7] Walia GS, Broyles JM, Christensen JM, Lo AY, Rochlin DH, Daily FF, Shridharani SM, Sacks JM (2017). Pedicled anterolateral thigh flaps for salvage reconstruction of complex abdominal wall defects. Clin Surg.

[8] Krpata DM, Stein SL, Eston M, Ermlich B, Blatnik JA, Novitsky YW, Rosen MJ (2013). Outcomes of simultaneous large complex abdominal wall reconstruction and enterocutaneous fistula takedown. Am J Surg.

[9] Lin SJ, Butler CE (2010). Subtotal thigh flap and bioprosthetic mesh reconstruction for large, composite abdominal wall defects. Plast Reconstr Surg.

[10] Song YG, Chen GZ, Song YL (1984). The free thigh flap: a new free flap concept based on the septocutaneous artery. Br J Plast Surg.

[11] Kimata Y, Uchiyama K, Sekido M, Sakuraba M, Iida H, Nakatsuka T, Harii K (1999). Anterolateral thigh flap for abdominal wall reconstruction. Plast Reconstr Surg.

[12] Song Z, Yang D, Yang J, Nie X, Wu J, Song H, Gu Y (2018). Abdominal wall reconstruction following resection of large abdominal aggressive neoplasms using tensor fascia lata flap with or without mesh reinforcement. Hernia J Hernias Abdom Wall Surg.

[13] Shih PK (2018). Feasibility of pedicled anterolateral thigh flap with tensor fascia lata and vastus lateralis for difficult abdominal wall closure. Hernia J Hernias Abdom Wall Surg.

[14] Kayano S, Sakuraba M, Miyamoto S, Nagamatsu S, Taji M, Umezawa H, Kimata Y (2012). Comparison of pedicled and free anterolateral thigh flaps for reconstruction of complex defects of the abdominal wall: review of 20 consecutive cases. J Plast Reconstr Aesthet Surg.

[15] Ali F, Safawi EB, Zakaria Z, Basiron N (2013). Abdominal wall reconstruction after resection of an enterocutaneous fistula with an island pedicled anterolateral thigh perforator flap Case report. Clin Ter.

[16] Chang SH, Hsu TC, Su HC, Tung KY, Hsiao HT (2010). Treatment of intractable enterocutaneous fistula with an island pedicled anterolateral thigh flap in Crohn’s disease—case report. J Plast Reconstr Aesthet Surg.

[17] Lambe G, Russell C, West C, Kalaiselvan R, Slade DA, Anderson ID, Watson JS, Carlson GL (2012). Autologous reconstruction of massive enteroatmospheric fistulation with a pedicled subtotal lateral thigh flap. Br J Surg.

[18] Pruimboom T, Schols RM, Qiu SS, van der Hulst R (2018). Potential of near-infrared fluorescence image-guided debridement in trauma surgery. Case Reports Plast Surg Hand Surg.

[19] Cornelissen AJM, van Mulken TJM, Graupner C, Qiu SS, Keuter XHA, van der Hulst R, Schols RM (2018). Near-infrared fluorescence image-guidance in plastic surgery: a systematic review. Eur J Plast Surg.

[20] Federative Committee on Anatomical Terminology (FCAT) (1998). Terminologia anatomica.

[21] Visschers RG, van Gemert WG, Winkens B, Soeters PB, Olde Damink SW (2012). Guided treatment improves outcome of patients with enterocutaneous fistulas. World J Surg.

[22] Atema JJ, Furnee EJ, Maeda Y, Warusavitarne J, Tanis PJ, Bemelman WA, Vaizey CJ, Boermeester MA (2017). Major complex abdominal wall repair in contaminated fields with use of a non-cross-linked biologic mesh: a dual-institutional experience. World J Surg.

[23] Zerbib P, Caiazzo R, Piessen G, Rogosnitzky M, Sequier C, Koriche D, Truant S, Boleslawski E, Chambon JP, Pruvot FR (2015). Outcome in porcine acellular dermal matrix reinforcement of infected abdominal wall defects: a prospective study. Hernia J Hernias Abdom Wall Surg.

[24] Rosen MJ, Krpata DM, Ermlich B, Blatnik JA (2013). A 5-year clinical experience with single-staged repairs of infected and contaminated abdominal wall defects utilizing biologic mesh. Ann Surg.

[25] Doussot A, Abo-Alhassan F, Derbal S, Fournel I, Kasereka-Kisenge F, Codjia T, Khalil H, Dubuisson V, Najah H, Laurent A, Romain B, Barrat C, Tresallet C, Mathonnet M, Ortega-Deballon P (2019). Indications and outcomes of a cross-linked porcine dermal collagen mesh (permacol) for complex abdominal wall reconstruction: a multicenter audit. World J Surg.

[26] Kaufmann R, Timmermans L, van Loon YT, Vroemen J, Jeekel J, Lange JF (2019). Repair of complex abdominal wall hernias with a cross-linked porcine acellular matrix: cross-sectional results of a Dutch cohort study. Int J Surg (Lond, Engl).

[27] Sun WQ, Xu H, Sandor M, Lombardi J (2013). Process-induced extracellular matrix alterations affect the mechanisms of soft tissue repair and regeneration. J Tissue Eng.

[28] Phillips BT, Lanier ST, Conkling N, Wang ED, Dagum AB, Ganz JC, Khan SU, Bui DT (2012). Intraoperative perfusion techniques can accurately predict mastectomy skin flap necrosis in breast reconstruction: results of a prospective trial. Plast Reconstr Surg.

[29] Wormer BA, Huntington CR, Ross SW, Colavita PD, Lincourt AE, Prasad T, Sing RF, Getz SB, Belyansky I, Heniford BT, Augenstein VA (2016). A prospective randomized double-blinded controlled trial evaluating indocyanine green fluorescence angiography on reducing wound complications in complex abdominal wall reconstruction. J Surg Res.

[30] Blanco-Colino R, Espin-Basany E (2018). Intraoperative use of ICG fluorescence imaging to reduce the risk of anastomotic leakage in colorectal surgery: a systematic review and meta-analysis. Tech Coloproctol.

